# Out of the Shadows: Using Human Rights Approaches to Secure Dignity and Well-Being for People with Mental Disabilities

**DOI:** 10.1371/journal.pmed.0020071

**Published:** 2005-04-26

**Authors:** Alicia Ely Yamin, Eric Rosenthal

## Abstract

A human-rights-based policy on mental health is urgently needed, argue Yamin and Rosenthal. Around half a billion people suffer from a mental or behavioral disorder, yet only a small minority receive even the most basic treatment

Mental health is perhaps the most neglected area of health policy and programming. According to the 2001 World Health Report, “some 450 million people suffer from a mental or behavioral disorder, yet only a small minority of them receive even the most basic treatment” [1]. More than 40% of countries have no mental health policy and over 30% have no mental health program. Over 90% of countries have no mental health policy that includes children and adolescents [1]. According to the World Health Organization (WHO), mental and behavioral disorders are estimated to account for 12% of the global burden of disease, yet the mental health budgets of the majority of countries constitute less than 1% of their total health expenditures [1]. The relationship between disease burden and disease spending is clearly disproportionate.

Those few who do receive services often fare just as badly. Mental Disability Rights International (MDRI; Washington, D.C., United States), a human rights group dedicated to the promotion of rights of the mentally disabled, has documented how, in many countries, severely mentally disabled individuals become targets of stigma, discrimination, and other human rights abuses. Routinely, children and adults with mental disabilities are arbitrarily detained in psychiatric facilities, social care homes, orphanages and other closed institutions. Out of public view, they are subject to the most extreme forms of inhuman and degrading treatment experienced by any population (Figure 1). In Kosovo, MDRI learned that women were raped in psychiatric facilities in plain view of local staff and international humanitarian relief workers [2]. In Hungary and Paraguay, MDRI found people locked in cages [3,4]. In Turkey, Peru, and Bulgaria, MDRI investigators learned of a practice called “unmodified ECT”—the use of without any form of anaesthesia or muscle relaxants—a practice that is both painful and dangerous [5,6].

**Figure 1:**
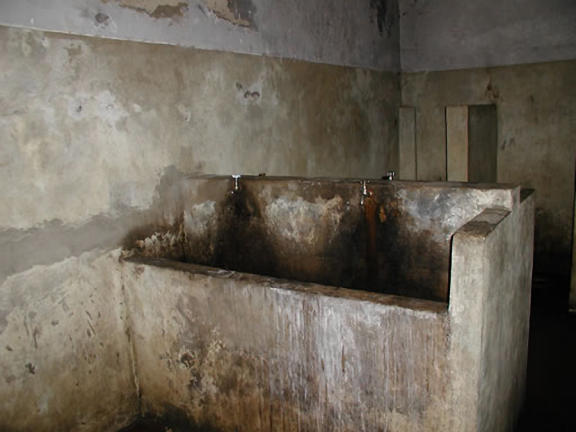
Bathroom in the Men's Chronic Ward of Larco Herrera Hospital, a Government-Supported Psychiatric Hospital in Lima, Peru An investigation of the men's chronic ward, by MDRI and Asociación Pro Derechos Humanos (a Peruvian nonprofit organization that works toward human rights), found that “conditions were stark, the bathrooms filthy, and severe regimentation denied patients' basic autonomy” [5]. (Photo: Mental Disability Rights International)

## Defining a Human Rights Approach to Mental Health Policy

The starting point for the development of a human-rights based policy on mental health is that mentally ill individuals are full human beings who are entitled to rights. Although seemingly obvious, in practice MDRI has found that the implications of these premises challenge predominant biomedical approaches to mental illness, as well as health services paradigms [7]. In a rights framework, “mental health needs” are not analyzed (as they are in many studies) in terms of the application of given diagnostic criteria in isolation from the social context that leads to use of the mental health sector, and mentally disabled persons are treated as more than patients who need services [7,8]. They have rights to exercise agency in their own lives and to participate as members of their communities and societies, and these rights trump other concerns such as general attitudes toward risk containment in society [9].

Thus, a human rights framework calls for changes that go beyond quality of care to include both legal and services reforms. Further, a human rights approach demands that we develop policies and take actions to end discrimination in the overall society that has a direct effect on the health and well-being of the mentally disabled.

## Legal Reform and Accountability

Suspicion of mental illness cannot mean untrammeled discretion to disregard due process concerns in detention. Whether or not ideological factors are at play, civil commitment laws must provide for minimum substantive and procedural protections that protect mentally ill individuals' fundamental agency [10]. This is often not the case. For example, MDRI found that civil commitment laws in Uruguay and Mexico allow commitment upon medical certification of “mental illness,” which MDRI found, in many cases, to be questionable. There are no requirements that a patient be dangerous or in need of psychiatric treatment. These laws do not require a right to counsel or a periodic review of commitment, as international law requires [11,12]. Thus, people who are found “mentally ill” can be deprived of their liberty indefinitely in these—and many other—countries.

Once mental health is construed in terms of human rights, all states are required, at a very minimum, to establish a normative framework consistent with international law [13]. Such a normative framework provides for procedural protections; it also provides for human rights oversight and remedies in the event of abuses. Mentally disabled persons have the same right to redress for violations of their fundamental rights as other people do [13,14,15]. Recourse may imply judicial remedies but in some cases a human rights ombudsman can be equally effective [16]. As mentally disabled individuals are often not in a position to avail themselves of remedies, proactive monitoring and enforcement is also necessary. In short, instituting legal reform, accountability procedures, and effective mechanisms to provide human rights oversight becomes a cornerstone of a human rights-based approach to mental health policy.

## Services Reform: Community Integration and Participation

Treating mentally ill people as full human beings implies that they have rights to participate in their communities and societies. This, in turn, calls for community integration and service system reform instead of programs that merely rebuild segregated institutions. The WHO also recognizes that it is important to provide treatment in the community, but its reasoning is largely utilitarian. The WHO argues that “community care has a better effect than institutional treatment on the outcome and quality of life of individuals with chronic mental disorders. Shifting patients from mental hospitals to care in the community is also cost-effective” [1]. From a human rights perspective, people are entitled to live in and receive care in the community not because it is more efficient, but because all human beings develop their identities within social contexts, and have rights to work and study, as well as be with family and friends.

People with mental disabilities are often denied the right to work outside the home, to marry or have children

A rights-based approach calls not only for the location of care in the community, but also for the transfer of planning and decision-making power to the individuals and communities that the health system is supposed to serve. In this case, consumers and family members must be integrally involved in the policy-making and programming decisions [13,17,18,19].

MDRI has repeatedly found that funds are misdirected toward rebuilding psychiatric institutions and orphanages. Further, international institutions often undermine rights-based approaches to policy. MDRI has documented how European governments, development banks and international humanitarian relief organizations fund projects to build new psychiatric institutions and orphanages throughout the Americas and Eastern Europe, rather than focusing on community care [9].

## Non-Discrimination: Within and beyond the Health Sector

Nondiscrimination is the most fundamental tenet in human rights. Under international law, discrimination need not be intentional nor de jure (in law) to constitute a violation of various relevant treaties, but merely needs to have the “effect of nullifying or impairing the equal enjoyment or exercise” of rights (paragraph 11 of [20]).

MDRI has found that discrimination against people with mental disabilities is pervasive, and takes many forms. In Peru, for example, the public health insurance scheme does not cover mental disorders [5]. However, it is critical to recognize that discrimination outside the health sector also affects well-being. MDRI has seen that children with intellectual disabilities are denied equal access to education in Peru. They are placed in programs that in effect warehouse them and assume that they are unable to learn [5]. As adults, people with mental disabilities are often denied the right to work outside the home, to marry or have children, or to take part in the religious and social activities that define people as adult members of society [21].

A human rights approach to mental health policy demands that special attention be placed on remedying such inequities—both within and beyond the health sector—which affect the physical, mental, and social well-being of persons with mental disabilities [22]. Such an approach depends upon multisectoral strategies including education, housing, and work, and establish that people with mental disabilities are full citizens.

## Conclusions and Reflections on First Steps

MDRI works through a human rights framework that links the improvement of mental health services with broader questions of social justice and nondiscrimination relating to the full spectrum of rights set out in international instruments [13,14]. As an immediate first step, all states—regardless of resources—can develop national mental health policies and plans of action with measurable targets, which provide for open public discussion [13,23]. Devising a national policy is a precondition to creating rights-based programs that address the multivalent problems faced by persons with mental disability [1]. Stakeholder participation in the process affirms that mentally disabled people are rights-worthy [13,17].

Both international agencies and professional associations can play critical roles in providing technical assistance to countries to develop rights-based national mental health policies [1,4]. Bilateral and multilateral donors should encourage such rights-based policies through their funding prerogatives.

## Abbreviations

MDRI: Mental Disability Rights International
WHO: World Health Organization
